# Role of MicroRNAs in Host Defense against Infectious Bursal Disease Virus (IBDV) Infection: A Hidden Front Line

**DOI:** 10.3390/v12050543

**Published:** 2020-05-14

**Authors:** Jiaxin Li, Shijun J. Zheng

**Affiliations:** 1 Key Laboratory of Animal Epidemiology of the Ministry of Agriculture, College of Veterinary Medicine, China Agricultural University, Beijing 100193, China; b20183050414@cau.edu.cn; 2 College of Veterinary Medicine, China Agricultural University, Beijing 100193, China

**Keywords:** infectious bursal disease virus, microRNAs, infection

## Abstract

Infectious bursal disease (IBD) is an acute, highly contagious and immunosuppressive avian disease caused by infectious bursal disease virus (IBDV). In recent years, remarkable progress has been made in the understanding of the pathogenesis of IBDV infection and the host response, including apoptosis, autophagy and the inhibition of innate immunity. Not only a number of host proteins interacting with or targeted by viral proteins participate in these processes, but microRNAs (miRNAs) are also involved in the host response to IBDV infection. If an IBDV–host interaction at the protein level is taken imaginatively as the front line of the battle between invaders (pathogens) and defenders (host cells), their fight at the RNA level resembles the hidden front line. miRNAs are a class of non-coding single-stranded endogenous RNA molecules with a length of approximately 22 nucleotides (nt) that play important roles in regulating gene expression at the post-transcriptional level. Insights into the roles of viral proteins and miRNAs in host response will add to the understanding of the pathogenesis of IBDV infection. The interaction of viral proteins with cellular targets during IBDV infection were previously well-reviewed. This review focuses mainly on the current knowledge of the host response to IBDV infection at the RNA level, in particular, of the nine well-characterized miRNAs that affect cell apoptosis, the innate immune response and viral replication.

## 1. Introduction

Infectious bursal disease (IBD), also known as Gumboro disease, is an acute, highly contagious and immunosuppressive avian disease caused by infectious bursal disease virus (IBDV). The disease first broke out in the Gumboro town of Delaware in USA and was recorded as a new disease by Cosgrove in 1962 [1,2]. Since then, IBDV has been widely distributed around the world [3,4]. Chickens especially young chicks at the age of 3-6 weeks are the most susceptible to IBDV infection [5]. In addition to the morbidity of infected chickens, IBDV infection mainly damages the bursa of Fabricius [6], the central immune organ of chickens for the development and maturation of B lymphocytes, directly causing a rapid, progressive loss of B lymphocytes in the bursa, spleen, and peripheral blood by apoptosis, leading to immunosuppression in survived chickens [7]. In addition, IBDV also suppresses innate immune responses by inhibiting the type I interferon (I-IFN) expression and melanoma differentiation-associated gene-5 (MDA5, a pattern recognition receptor)-dependent signaling pathways [8]. Thus, the survival of IBDV-infected chickens has a compromised immune response to secondary or complicated infections or subsequent vaccination against other diseases, such as Avian Influenza(AI), Newcastle Disease(ND) [9] and Infectious Bronchitis(IB) [10]. Despite the availability and application of IBDV vaccines in poultry worldwide, the continued emergence of new variant IBDV (vIBDV) and new very virulent IBDV (vvIBDV) still threatens the poultry industry around the world [11,12]. More effective vaccines are and will be in great demand for the control of IBD. Therefore, a complete understanding of the pathogenesis of IBDV infection will be of great help to the development of novel vaccines.

MicroRNAs (miRNAs) were first discovered in C. elegans in the early 1990s [13,14], but their biological functions were gradually discovered in the early 21st century [15,16]. Currently, it is known that miRNAs are epigenetically regulated and involved in cell proliferation, differentiation, senescence and apoptosis, and are associated with various diseases, including tumors, cancers and viral infections [17,18]. Thus, the elucidation of the biological activities of miRNAs and the mechanism of their actions will be helpful for disease control and drug development. The effect of miRNAs on viral replication during infection is complex. On one hand, some host miRNAs may act as antiviral factors inhibiting viral replication. For example, during IBDV infection, chicken microRNAs (gga-miR)-130b-3p [19], gga-miR-454-3p [20], gga-miR-155-5p [21] and gga-miR-27b-3p [22] act as antiviral factors suppressing IBDV replication. On the other hand, viruses may take advantage of the host miRNAs to enhance their replication and infection. For example, gga-miR-9-3p [23], gga-miR-2127-3p [24], gga-miR-142-5p [25] and gga-miR-16-5p [26] inhibit host defense and favor IBDV replication. It seems that host miRNAs may act either as an antiviral weapon or can be manipulated by viruses to favor their survival. Here, we summarize the recent findings that focus on the functions of gga-miRNAs and the underlying mechanisms during IBDV infection, providing new insights into the elucidation of the pathogenesis of IBDV infection and references for the investigation of other pathogens.

## 2. Virus Characteristics

IBDV is an immunosuppressive avian pathogen containing a non-enveloped, icosahedral capsid with a diameter of about 60 nm [2,27]. It belongs to the genus *Avibirnavirus* in the family Birnaviridae, harboring bi-segmented double-stranded RNA (dsRNA) genomes, namely segment A (3.2 kb) and segment B (2.8 kb) [28]. Segment A contains two partially overlapping open reading frames (ORFs) encoding a small nonstructural protein VP5 (17 kDa) [29] and a large polyprotein pVP2-VP4-VP3 (109 kDa). The pVP2-VP4-VP3 later undergoes a rapid self-proteolytic processing, yielding three polypeptides: the precursor of the capsid protein pVP2 (54 kDa) [30], the serine protease VP4 (25 kDa) [31] and the scaffold protein VP3 (28 kDa) [32]. The pVP2 is further processed into the mature form of VP2 by VP2 itself [33]. Segment B encodes only the RNA-dependent RNA polymerase protein (RdRp) VP1 (90 kDa), a structural protein linked with a viral genome and VP3 [34,35]. The genome of IBDV encodes only these five viral proteins, and each of them may perform multiple functions to make the virus a successful pathogen during evolution [36]. Upon IBDV infection, the host cells recognize the pathogen-associated molecular patterns (PAMPs, such as dsRNA in the case of IBDV) via pattern recognition receptors (PRRs, such as TLR3 and MDA-5) and mobilize all necessary resources to inhibit virus replication, while IBDV, the invader, employs viral proteins as weapons to fight against the host response. Thus, a life-and-death struggle between IBDV and the host always continues. 

## 3. The Battle between IBDV and Host

The war between pathogens and hosts will never end. It is well-known that IBDV infection induces apoptosis in target cells of the bursa of Fabricius [37,38,39], which subsequently leads to immunosuppression [5]. Apoptosis is generally considered as an important defense mechanism of host response against microbial infection, which can limit intracellular bug replication. However, the process of apoptosis occurring in IBDV-infected cells seems to be well controlled by the virus. In the early stage of IBDV infection, VP5 inhibits apoptosis by interacting with the p85α subunit of PI3K, supporting viral replication [40]. However, in the later stage of IBDV infection, VP5 induces apoptosis through an interaction with the voltage-dependent anion channel 2 (VDAC2), thereby promoting virus release and spread [41]. In addition, VP5, VDAC2 and the receptor of the activated protein kinase C1 (RACK1) form a complex that regulates apoptosis in virus-infected cells, inhibiting apoptosis and enhancing viral replication [42]. Thus, VP5 may play opposite roles in apoptosis in different stages of IBDV infection for the maximal benefit of viral growth. Furthermore, the IBDV VP2 protein induces apoptosis via the interaction and degradation of oral cancer overexpressed 1 (ORAOV1), a protein that acts as an anti-apoptotic molecule in host cells, enhancing viral replication [43]. A recent publication by our laboratory indicates that IBDV infection upregulates the expression of cellular gga-miR-16-5p in DF-1 cells, which enhances IBDV-induced apoptosis by directly targeting the cellular anti-apoptotic protein B-cell lymphoma 2 (Bcl-2), facilitating IBDV replication in host cells [26]. Thus, IBDV manipulates the apoptotic process in host cells at both the protein and RNA levels to favor its survival. 

In addition to apoptosis, another consequence of IBDV infection is immunosuppression in the host. In general, avian dsRNA virus, once inside the cells, will trigger an innate immune response in the host cells via the engagement of cellular chicken MDA5 (chMDA5) by viral RNA [44], and then it activates the downstream transcription factor IRF3/IRF7, inducing the production of the type I interferon (I-IFN, which subsequently binds to the interferon receptor, initiating the next antiviral signaling pathway). However, in the case of IBDV infection, it was found that the VP3 protein strongly binds with MDA5 to block its recognition of dsRNA, a PAMP of IBDV, thereby significantly inhibiting the I-IFN expression [8]. A recent report shows that IBDV genomic dsRNA selectively binds to the N-terminal moiety of Staufen1 (STAU1), a host cellular RNA-binding protein, promoting viral replication via attenuating an MDA5-dependent β interferon induction [45]. In addition, the VP3 protein interacts with both the ribosomal protein L18 (chRPL18) and chicken double-stranded RNA-activated protein kinase (chPKR), promoting viral replication [46], and it can also interact with chicken eukaryotic translation elongation factor 1α (cheEF1α), promoting the activity of IBDV polymerase [47]. Furthermore, besides VP3, IBDV VP4 acts as an interferon suppressor and promotes viral replication by inhibiting the K48-linked ubiquitylation of glucocorticoid-induced leucine zipper (GILZ) in the host cells [48,49]. Thus, it seems that IBDV employs varied strategies to evade host anti-virus defense through the interaction of VP3 or VP4 with its corresponding cellular partners, suppressing the innate immune response of the host cells and facilitating viral replication. IBDV infection also affects the expressions of gga-miRNAs, and it was found that gga-miR-9-3p [23], gga-miR-2127-3p [24], and gga-miR-142-5p [25] inhibit host defense and favor viral replication. Therefore, IBDV attempts in various ways to fight against the host cells for survival via evading or inhibiting the innate immune response at both the protein and RNA levels. Host cells are not totally passive during IBDV infection. Some intracellular factors can also affect viral replication. It was found that cellular eukaryotic translational initiation factor 4AII (eIF4AII) interacts with the VP1 protein, inhibiting IBDV replication in the host cells [50]. At the RNA level, host cells express cellular gga-miR-130b-3p [19], gga-miR-454-3p [20], gga-miR-155-5p [21] and gga-miR-27b-3p [22] in response to IBDV infection. These miRNAs, as antivirus factors, suppress IBDV replication via enhancing the I-IFN expression or a direct targeting of the viral genome. 

IBDV may also take advantage of some cellular factors or mechanisms for its own use. It was found that the voltage-dependent anion channel 1 (VDAC1) interacts with the IBDV ribonucleoprotein complex containing VP1, VP3 and dsRNA [51], and plays a critical role in mediating viral replication and transcription during the virus life cycle [35,52,53]. Interactions of VDAC1 with VP3 and VP1 help to stabilize the interaction between VP3 and VP1, promoting the IBDV polymerase activity. A recent publication shows that VP1 is efficiently modified by the K63-linked ubiquitin chain at the K751 residue in the C terminus as well as the small ubiquitin-like modifier 1 (SUMO1) at its 404I and 406I residues [54,55]. Ubiquitination and sumoylation of VP1 significantly enhanced its polymerase activity and maintained the stability of the polymerase VP1. Thus, the interaction between IBDV and host cells is a complex process, involving multiple factors and mechanisms of biological activities from protein to RNA levels.

## 4. MiRNA Biogenesis and Nomenclature 

MicroRNAs are currently the best-described non-coding RNAs (ncRNAs) that follow a specific biogenesis pathway. The biogenesis of miRNAs involves several processing steps and is well-reviewed in the literature [56,57,58]. Briefly, miRNAs are first transcribed by the RNA polymerase II as a long (>1kb) primary miRNA (pri-miRNA) that contains a local stem-loop structure, typically consisting of a 33-35 nt stem, a terminal loop and 5ʹ and 3ʹ single-stranded RNA (ssRNA) flanking sequences. Then these miRNAs are processed into 70 nt precursor miRNAs (pre-miRNA) in the nucleus by the microprocessor complex, which consists of the RNase III enzyme Drosha, the RNA-binding protein, and the DiGeorge syndrome critical region 8 (DGCR8). Thereafter, the pre-miRNA hairpins are exported into the cytoplasm via exportin 5 (Exp5) and a GTP-binding nuclear protein (RanGTP), then recognized by another RNase III endonuclease, Dicer, which serves as a molecular ruler and cleaves off the hairpin head approximately 22 nt from the 3′ end, thereby generating a 22 nt miRNA duplex. Subsequently, a mature miRNA is produced and assembled into the RNA-induced silencing complex (RISC) by the Ago protein facilitated by the Hsc70/Hsp90 chaperones in an ATP-dependent manner. The miRNA directs the RISC to target the mRNA sequences by complementarity, inducing mRNA translational repression, mRNA deadenylation and mRNA decay.

The nomenclature of microRNAs has a clear form which are described in detail on the miRBase website (http://www.mirbase.org/help/nomenclature.shtml), like “gga-miR-155”. The prefix signifies the organism. Thus, “gga” refers to chicken, “hsa” refers to human, “ssc” refers to pig and “mmu” refers to mouse. The middle “miR” is designated the mature miRNA when the “R” is capitalized, otherwise the “mir” indicates different meanings, and may be the miRNA precursor or the miRNA gene, or may be the predicted stem-loop portion of the primary transcript. The numbers are assigned sequentially. Homologous miRNAs share the same number, regardless of organism. For paralogous miRNAs from distinct precursor sequences and genomic loci, miRNAs are given a numbered suffix when expressing identical mature sequences (for example, gga-miR-16-1 and gga-miR-16-2). A letter suffix is given when the derived mature miRNAs are closely related but contain differences (for example, gga-miR-130b and gga-miR-130c). Generally, not both strands produced in a miRNA gene are active. Formerly, one of the two mature products of a miRNA gene is called the “mature strand” or “guide strand”, and is considered to be the miRNA because it is more highly expressed, while the opposite strand is called the “passenger strand” or “star strand” (miR^*^), since it is less expressed and thought to be inactive (for example, gga-miR-9 and gga-miR-9^*^). Later, it was found that alternative strands however, can be differentially expressed in different tissues, developmental stages, pathological states or species, requiring an inversion of the miR/miR^*^ annotation to reflect the expression variations if the miR^*^ strand is functionally active. For this reason, the “miR/miR^*^” symbolism has been replaced with the unbiased “5p/3p” strand annotation to indicate the position of the strand in the pre-miRNA hairpin independently of its expression status in any given dataset since release 17 in miRBase (names like gga-miR-142-5p and gga-miR-142-3p) [59]. As a consequence, in this article, we followed the above naming rules and used the latest miRNA names instead of incomplete names in some references by checking the sequence of these miRNAs in the literature and the miRBase database one by one.

## 5. Roles of MiRNAs in Host Response to IBDV Infection

Since the first discovery of EBV-encoded miRNA [60], some viruses, including several avian viruses, were found to encode miRNAs, such as Marek’s disease virus (MDV), herpesvirus of turkeys (HVT) and infectious laryngotracheitis virus (ILTV) [61]. Virus-encoded miRNAs can target both the cellular and viral mRNAs to promote an intracellular environment favorable to the completion of the viral life cycle. As the genome of IBDV does not encode miRNAs, only the host-encoded miRNAs will be discussed hereafter. It was reported that during IBDV infection of DF-1 cells, a total of 296 miRNAs were found to be involved in major antiviral pathways as demonstrated by a high-throughput sequencing assay [19]. Among those miRNAs, 214 were predicted to be engaged in a JAK-STAT signaling pathway, 207 in a Toll-like receptor (TLR)-mediated signaling pathway, 164 in an Retinoic acid-inducible gene I (RIG-I)-like helicase receptor (RLR)-mediated signaling pathway and 244 in a cytokine–cytokine receptor signaling pathway [19]. It was also found that 18 miRNAs were identified and significantly altered (of which, 11 were downregulated and 7 were upregulated) in 991 conserved miRNAs in IBDV-stimulated chicken dendritic cells (DCs) [62]. Using miRDB and TargetScan, 2317 target genes were forecasted for the differentially expressed miRNAs in IBDV-stimulated chicken DCs. These potential target genes were involved in the intracellular signaling cascade, protein localization and phospho-metabolic processes, as well as the mitogen-activated protein kinase (MAPK), mTOR and neurotrophin signaling pathways by GO categorization, KEGG and BIOCARTA pathway analyses [62]. It is highly possible that a large number of gga-miRNAs participate in the cellular immune response and metabolism post-IBDV infection.

Up to now, nine gga-miRNAs have been reported to play important roles in the host–IBDV interaction, including gga-miR-130b-3p, gga-miR-454-3p, gga-miR-155-5p, gga-miR-21-5p, gga-miR-27b-3p, gga-miR-2127-3p, gga-miR-142-5p, gga-miR-9-3p and gga-miR-16-5p. These gga-miRNAs, based on their effects on viral infection, can be divided into two groups: groups one and two. Group one includes five gga-miRNAs (gga-miR-130b-3p, gga-miR-454-3p, gga-miR-155-5p, gga-miR-21-5p and gga-miR-27b-3p) exerting antiviral effects on IBDV infection, while group two is composed of four miRNAs (gga-miR-2127-3p, gga-miR-142-5p, gga-miR-9-3p and gga-miR-16-5p) promoting IBDV replication by inhibiting host defense. The current understandings of the roles of these miRNAs in the host response to IBDV infection will be described below.

### 5.1. MiRNAs Inhibiting IBDV Infection

Up to now, five gga-miRNAs (gga-miR-130b-3p, gga-miR-454-3p, gga-miR-155-5p, gga-miR-21-5p and gga-miR-27b-3p) have been found to inhibit IBDV infection. In addition to these chicken miRNAs, their mammalian counterparts will also be discussed below where necessary. 

#### 5.1.1. gga-miR-130b-3p

miR-130b-3p, belonging to the miR-130/301 family, is encoded by human chromosome 22 and chicken chromosome 15 (https://www.ncbi.nlm.nih.gov/gene/?term=mir-130b) and is involved in different human physiological activities and cancers [63,64]. The dysregulation of miR-130b-3p is directly implicated in angiogenesis, which appears to be a significant process during cancer metastasis [65]. Recent studies have shown that hsa-miR-130b-3p acts as a tumor suppressor, inhibiting cancer angiogenesis by specifically targeting tumor necrosis factor-α (TNF-α), thereby suppressing the nuclear factor-κB/vascular endothelial growth factor-A (NF-κB/VEGFA) signaling pathway [66]. 

**Figure 1:**
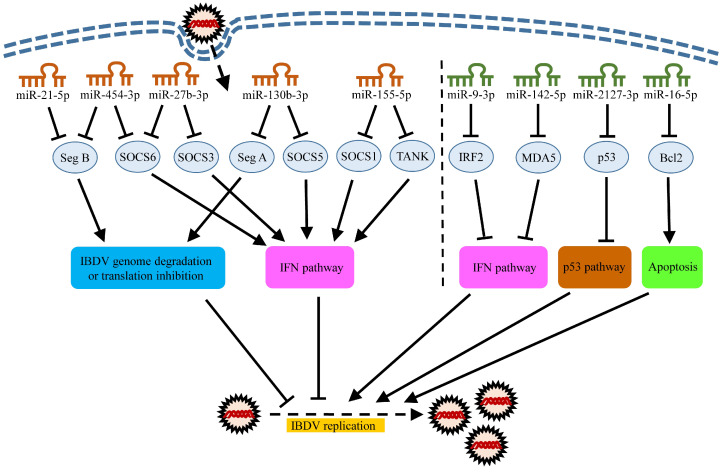
Schematic diagram of the roles of gga-miRNAs in host response to infectious bursal disease virus (IBDV) infection. IBDV infection affects the expression of miRNAs in host cells. These miRNAs promote or inhibit IBDV replication by directly targeting the IBDV’s genome or the mRNA of the host genes involved in the immune signaling pathway. Seg A: IBDV genome segment A; Seg B: IBDV genome segment B; SOCS1: suppressor of cytokine signaling 1; SOCS3: suppressor of cytokine signaling 3; SOCS5: suppressor of cytokine signaling 5; SOCS6: suppressor of cytokine signaling 6; TANK: TRAF family member-associated NF-κB activator; IRF2: interferon regulatory factor 2; MDA5: melanoma differentiation-associated gene 5; Bcl2: B-cell lymphoma 2, an anti-apoptosis protein; p53: a tumor suppressor with a mass of around 53 kDa; IFN: interferon.

The expression of gga-miR-130b-3p in DF-1 cells is markedly increased after IBDV infection, and gga-miR-130b-3p suppresses IBDV replication via directly targeting both the viral specific gene sequence in genomic segment A and suppressors of cytokine signaling 5 (SOCS5) in host cells (Figure 1) [19]. SOCS5 is a member of the SOCS family, which consists of eight structurally similar proteins, including SOCS1-7 and the cytokine-induced SH2-containing proteins (CIS) [67]. The SOCS family proteins are well-known negative regulators for cytokine receptors’ signaling by inhibiting the JAK-STAT signaling transduction to prevent an excessive immune response [68]. They also play an important role in receptor tyrosine kinases’ (RTKs) signals [69]. The inhibition of the SOCS5 expression by gga-miR-130b-3p enhances the mRNA expressions and phosphorylation of STAT1, subsequently activating the I-IFN signaling pathway that plays a major role in the host antiviral response. Unfortunately, the binding site of gga-miR-130b-3p in the genome of very virulent IBDV (vvIBDV) has mutations, while it is relatively conserved in most classical and low-virulence IBDV strains [19], suggesting that some IBDV strains make clever use of its RNA polymerase’s lack of corrective function to evade the host’s immune response through its own mutation and evolution. 

In addition to IBDV, there are a few reports about the antiviral effects of miR-130b-3p on other viruses. It was reported that ssc-miR-130b-3p directly targets the 5′ untranslated region (UTR) of the porcine reproductive and respiratory syndrome virus (PRRSV) genome, exerting an antiviral effect, and the intranasal inoculation of piglets with ssc-miR-130b-3p partially protects pigs from the deadly challenge of HP-PRRSV strains [70], which provides a scientific basis for the treatment of viral infections using cellular miRNAs. hsa-miR-130b-3p is stimulated in the liver by 25-hydroxycholesterol (25-HC), an antiviral oxysterol secreted by interferon-stimulated macrophages and dendritic cells during Hepatitis C virus (HCV) infection, inhibiting HCV replication [71]. gga-miR-130b-3p inhibits the cell cycles of the Marek’s disease lymphoblastoid cell line MDCC-MSB1 by hindering the expression of two genes, matrix metallopeptidase 2 (MMP2) and matrix metallopeptidase 9 (MMP9), which are closely correlated to cell invasion [72]. However, Marek’s disease virus (MDV) inhibits the gga-miR-130b-3p expression by promoting the methylation level of the upstream promoter region of the gga-miR-130b-3p gene in MDV-infected tumorous spleens [72]. Thus, it seems that miR-130b-3p is not only a tumor suppressor, but also an antiviral factor of host cells. More efforts will be required to determine the potential value of miR-130b-3p as a drug for the treatment of viral diseases. 

#### 5.1.2. gga-miR-454-3p

The gene for miR-454-3p, located on human chromosome 17 or chicken chromosome 15 (https://www.ncbi.nlm.nih.gov/gene/?term=mir-454), was found to modulate the tumorigenesis and progression of several malignancies [73,74,75]. Similar to gga-miR-130b-3p, gga-miR-454-3p suppresses IBDV replication through two mechanisms. On one hand, it directly degrades or inhibits the viral protein translation of the IBDV genome via targeting IBDV genomic segment B (encoding VP1 protein). Of note, the target sequence (TGCACT) of gga-miR-454-3p on segment B is relatively conserved in classical and low-virulent strains such as IBDV Lx, Cu-1 and B87 strains, but this region of IBDV has evolved to TGCACC/G in vvIBDV such as IBDV Gx, HK46 and UK661 strains, which cannot complement with the seed region of gga-miR-454-3p, thus allowing vvIBDV to avoid being targeted by gga-miR-454-3p [20]. The variation of the binding sites of both gga-miR-454-3p and gga-miR-130b-3p on the genome of the vvIBDV strain at least partially explains the difference in pathogenicity between the hypovirulent IBDV and vvIBDV strains. On the other hand, gga-miR-454-3p increases the expression of IFN-β by targeting the suppressors of cytokine signaling 6 (SOCS6) in DF-1 cells, enhancing the innate immune response of the host cells to IBDV infection (Figure 1) [20]. Recently, it was reported that hsa-miR-454-3p directly targets SOCS6 in human liver cell lines and promotes the expansion of liver tumor-initiating cells [73], suggesting a wide interaction between miR-454-3p and SOCS6. However, unlike gga-miR-130b-3p, the expression level of gga-miR-454-3p in DF-1 cells is significantly reduced after IBDV infection [20], suggesting that gga-miR-454-3p can hardly exert an antiviral activity because IBDV infection inhibits the gga-miR-454-3p expression in host cells. It is worthwhile investigating into the mechanisms underlying the IBDV-induced inhibition of the gga-miR-454-3p expression. Currently, there is no report available regarding the effect of miR-454-3p on host defense against other viruses.

#### 5.1.3. gga-miR-155-5p

miR-155-5p is a typical multifunctional miRNA, located on human chromosome 21 and chicken chromosome 1 (https://www.ncbi.nlm.nih.gov/gene/?term=mir-155). It involves multiple roles including the initiation and development of tumors and cancers, the modulation of the immune response and participation in antiviral responses [76]. The abnormal expression of miR-155-5p is common in several human cancers, malignancies [77,78] and avian oncogenic viruses such as MDV, avian leukosis virus (ALV) and reticuloendotheliosis virus (REV) [79]. In addition, miR-155-5p plays a critical role in both innate and adaptive immune responses by regulating the differentiation and function of many kinds of immune cells, including CD4^+^ CD25^+^ regulatory T (Treg)/T helper (Th)17 cell [80], macrophage [81], CD4^+^ T cells [82] and CD8^+^ T cells [83]. A deficiency of hsa-miR-155-5p in CD4^+^ T cells affects their activation, proliferation and cytokine production in vitro and in vivo during vesicular stomatitis virus (VSV) infection [84]. CD8^+^ T cells lacking miR-155-5p can develop normally in a physiologically homeostatic condition but show impaired proliferation and survival ability during both virus infection and cancer [85]. Hence, miR-155-5p emerges as an irreplaceable molecule in various cellular processes for both humans and animals. 

Upon IBDV infection, the expression of gga-miR-155-5p is upregulated in a time- and dose-dependent manner, and it plays an antiviral role, enhancing the I-IFN expression via directly targeting SOCS1 and TANK (Figure 1) [21]. SOCS1 belongs to the SOCS family that acts as a negative regulator for immune signaling and tumor suppressors [86,87]. TANK, also known as the TRAF family member-associated NF-κB activator, is an adapter protein blocking the TNF receptor-associated factor 2 (TRAF2) to bind to latent membrane protein 1 (LMP1) and inhibit the LMP1-mediated NF-κB activation [88,89]. It also negatively regulates the NF-κB activation through the deubiquitination of IKBKG or TRAF6 in response to interleukin-1-β stimulation or upon DNA damage [90]. Furthermore, although the homology of the amino acid sequence of SOCS1 between chicken and human is only about 60% and the effect of miR-155-5p on SOCS1 in mammalian cells was well established [91,92,93], through the comparative research, it was found that the human and chicken SOCS1 have the same 3’ UTR motifs (AGCAUUAA) recognized by miR-155-5p [21,91,92]. 

miR-155-5p is one of the most intensively studied miRNAs. There are quite a few papers about the roles of miR-155-5p in the host response to viral infection. It was reported that the REV strain T upregulates the gga-miR-155-5p expression in chicken embryo fibroblast (CEF) cells after infection, while gga-miR-155-5p facilitates cell survival by targeting the jumonji and AT-rich interaction domain containing 2 (JARID2/Jumonji), a cell cycle regulator and part of a histone methyltransferase complex [94]. Recently, it was found that gga-miR-155-5p could also target caspase-6 (an apoptosis executor) and FOXO3a (which induces cell cycle arrest in the G0/G1 phase) to inhibit apoptosis and accelerate the cell cycle, thus improving the viability of REV-infected CEFs [95]. In addition, hsa-miR-155-5p was also found to inhibit enterovirus 71’s (EV71) replication through the promotion of the I-IFN response by targeting the FOXO3/IRF7 pathway [96]. Conversely, miR-155-5p may act as a villain to reinforce viral infections. It was reported that the hsa-miR-155-5p-mediated hepatitis B virus (HBV) replication by promoting the SOCS1/Akt/mTOR axis induced autophagy [97]. In the MDV-transformed cell line MSB1, gga-miR-155-5p targets the retinoid acid receptor-related orphan receptor alpha (RORA), a tumor suppressor, regulating the proliferation, cell cycle, apoptosis and invasiveness of MSB1 cells [98]. Thus, miR-155-5p is a critical miRNA that plays dual roles in viral infections. Further investigation into the molecular mechanism underlying the function of miR-155-5p will be definitely required to unravel the role of miR-155-5p in host response to pathogenic infections. 

#### 5.1.4. gga-miR-21-5p

The gene transcribing miR-21-5p is located on human chromosome 17 and chicken chromosome 19 (https://www.ncbi.nlm.nih.gov/gene/?term=mir-21). miR-21-5p has been found to be a pro-oncogene by targeting multiple tumor suppressor genes in both human and chicken, including programmed cell death 4 (PDCD4), phosphatase and tensin homolog (PTEN) [99,100,101]. gga-miR-21-5p was upregulated in the bursal cells of IBDV-infected specific pathogen free (SPF) chickens [102]. Using a lentiviral vector to establish a DF-1 cell line stably expressing gga-miR-21-5p (DF-miR-21-5p), Wang et al. found that the replication of IBDV significantly decreased in DF-miR-21-5p cells compared with that of controls, and that gga-miR-21-5p suppressed replication of IBDV through inhibiting the VP1 protein translation, not an mRNA degradation (Figure 1). Interestingly, the 22 nt targeted by gga-miR-21-5p in IBDV VP1 was not only present in the IBDV B87 strain used in Wang’s experiment, but was also conserved in at least 30 other strains [103], suggesting a broad-spectrum and conservation of the gga-miR-21-5p inhibition of IBDV infection.

miR-21-5p may have varied effects on different viruses. It was reported that the expression of ssc-miR-21-5p was upregulated in PK-15 cells after pseudorabies virus (PRV) infection, which inhibited the replication of the PRV by directly targeting chemokine (C-X-C motif) ligand 10 (CXCL10), also named interferon-γ inducible protein-10 (IP-10) [104]. On the contrary, hsa-miR-21-5p promoted the replication of dengue virus (DENV) subtype 2 in HepG2 cells [105]. Besides, hsa-miR-21-3p can repress the expression of histone deacetylase-8 (HDAC8) by targeting its 3’ UTR, promoting influenza A virus (IAV) replication, whereas host defenses act against IAV through the downregulation of hsa-miR-21-3p [106].

#### 5.1.5. gga-miR-27b-3p

The gene transcribing miR-27b-3p is located on human chromosome 9 and chicken chromosome Z (https://www.ncbi.nlm.nih.gov/gene/?term=mir-27b). Most of the research on miR-27b-3p is focused on its oncogenic and anti-cancer activity in humans [107,108]. However, the antiviral activity it exhibits deserves attention. A recent publication showed that IBDV infection upregulated the gga-miR-27b-3p expression by the demethylation of the pre-mir-27b promoter region [22]. Similar to the miRNAs as described above, gga-miR-27b-3p enhanced the mRNA expressions of STAT1, STAT3 and STAT6, increased the phosphorylation of STAT1 and enhanced the I-IFN expression via targeting two cellular suppressors of SOCS3 and SOCS6, suppressing IBDV replication in DF-1 cells (Figure 1) [22]. Likewise, ssc-miR-27b-3p significantly decreased the PRRSV replication in MARC-145 cells and porcine alveolar macrophages (PAMs) [109]. In the murine cytomegalovirus-infected mouse cell line, both mmu-miR-27b-3p and mmu-miR-27a-3p (a close homolog of mmu-miR-27b-3p (20/21 nucleotide identity)) exert an antiviral function upon over-expression [110]. Furthermore, it was demonstrated that ssc-miR-27b-3p attenuates apoptosis induced by transmissible gastroenteritis virus (TGEV) infection via targeting the runt-related transcription factor 1 (RUNX1) that regulates the Bax expression and the activities of caspase-3 and caspase-9 in TGEV-infected PK-15 cells [111].

As described above, these gga-miRNAs (gga-miR-130b-3p, gga-miR-454-3p, gga-miR-155-5p, gga-miR-21-5p and gga-miR-27b-3p) exert antiviral effects on IBDV infection largely by directly targeting the viral genome to inhibit viral replication or by targeting the negative regulators of the immune signaling pathways to enhance the innate immunity against viral infections (Figure 1).

### 5.2. miRNAs Promoting IBDV Replication

Up to now, four chicken miRNAs (gga-miR-2127-3p, gga-miR-142-5p, gga-miR-9-3p and gga-miR-16-5p) have been characterized to promote IBDV replication. The current understandings of the roles of these miRNAs including their mammalian counterparts in viral infections will be described below.

#### 5.2.1. gga-miR-2127-3p

miR-2127-3p has been only found in chickens so far, and it is located on chicken chromosome 1 (http://www.mirbase.org/cgi-bin/mirna_entry.pl?acc=MI0010732). It was reported that the expression of gga-miR-2127-3p was significantly upregulated in IBDV-infected DF-1 cells compared with that of the mock-infected controls, and this upregulation facilitated IBDV replication and dampened the chicken p53 (chp53) expression by targeting its 3’ UTR, attenuating a chp53-mediated anti-IBDV response (Figure 1) [24]. Similarly, new data confirmed that gga-miR-2127-3p was upregulated and targeted p53 during the H9N2 subtype avian influenza virus (AIV) infection, promoting viral replication [112]. In addition, the expression of gga-miR-2127-3p was also upregulated in both DF-1 cells and livers of 10-week-old chickens post-infection with subgroup J avian leukosis virus (ALV-J) [113,114]. Although the exact mechanism is not clear, it is speculated that gga-miR-2127-3p may play a certain role in the process of ALV-induced tumorigenesis.

#### 5.2.2. gga-miR-142-5p

Due to the lack of RIG-I in chickens [115], chicken MDA5 (chMDA5) is a key pattern recognition receptor (PRR) in chicken that recognizes viral RNAs and initiates the innate immune response to RNA virus infection [44]. It was reported that gga-miR-142-5p dampened chMDA5 by directly targeting the chMDA5 3′ UTR, attenuated the IRF7 signaling and favored the replication of IBDV in DT40 cells (Figure 1) [25]. However, it is unclear whether the expression of gga-miR-142-5p in host cells was affected by IBDV infection. If the gga-miR-142-5p expression was upregulated by IBDV infection, it was possible that some mechanism in the host cells was usurped by IBDV to enhance the expression of gga-miR-142-5p for its survival. Conversely, if the gga-miR-142-5p expression was downregulated due to IBDV infection, the innate response in host cells against IBDV infection should be enhanced.

miR-142-5p also plays a role in the mammalian immune response to other virus infection. It was found that ssc-miR-142-5p promoted the replication of porcine hemagglutinating encephalomyelitis virus (PHEV) by binding to the mRNA of unc-51-like-kinase1 (Ulk1), which controls axon outgrowth and dendrite formation, negatively regulating neuronal morphogenesis in mice with PHEV infection [116]. In addition, Rotavirus (RV)-induced hsa-miR-142-5p blocked host-mediated early apoptosis by targeting the non-canonical transforming growth factor beta (TGFβ), helping RV replication [117]. More efforts will be required to determine the role of miR-142-5p in host response to pathogenic infections.

#### 5.2.3. gga-miR-9-3p

The miR-9 gene family plays a pivotal role in the metastasis and angiogenesis of multiple cancer cells in humans and serves as a critical regulator of organ development and function in neurogenesis and myogenesis [118,119]. miR-9-3p, also known as miR-9^*^, is the lower-expressed miRNA in the two arms of the pre-mir-9 hairpin structure. Likewise, IBDV infection induces a significant upregulation of the gga-miR-9-3p expression in the bursa of Fabricius in SPF chickens, which negatively regulates the IBDV-triggered I-IFN production by directly targeting chicken interferon regulatory factor 2 (IRF2) (Figure 1) [23]. This finding has added to the understanding of the IBDV-induced suppression of interferon production in chickens. Furthermore, influenza A virus (IAV) hijacks has-miR-9-5p to benefit the IAV replication through targeting monocyte chemoattractant protein 1-induced protein 1 (MCPIP1), a PIN-like RNase inhibiting the IAV M and NP genes’ expressions and progeny production [120]. Moreover, the human coronavirus OC43 nucleocapsid protein can bind to has-miR-9-5p, preventing has-miR-9-5p from negatively regulating NF-κB, and enhancing the activation of NF-κB during viral infection [121]. Thus, the miR-9 family plays both negative and positive roles in host response to viral infections.

#### 5.2.4. gga-miR-16-5p

miR-16 is regarded as a tumor suppressor by inhibiting the proliferation and promoting apoptosis of human cancer cells [122]. It also plays anti-inflammatory roles in many inflammatory diseases [123]. miR-16 includes two paralogous members: mir-16-1 and mir-16-2. The gene transcribing mir-16-1 is located on human chromosome 13 or chicken chromosome 1 (https://www.ncbi.nlm.nih.gov/gene/?term=mir-16-1), while the mir-16-2 gene on human chromosome 3 or chicken chromosome 9 (https://www.ncbi.nlm.nih.gov/gene/?term=mir-16-2). miR-16-5p is the mature form of mir-16-1/mir-16-2. Recently, we have shown that IBDV infection upregulates the expression of gga-miR-16-5p via demethylating the promoter region of gga-mir-16-2 instead of gga-mir-16-1, and gga-miR-16-5p enhances IBDV-induced apoptosis in DF-1 cells by directly targeting cellular B-cell lymphoma-2 (Bcl2), a pivotal anti-apoptosis protein, facilitating IBDV replication (Figure 1), which supports the previous publication by others that both human and chicken miR-16-5p could directly target Bcl2 to induce apoptosis [124,125]. Apoptosis in host cells may have dual effects on viral survival, depending on the timing of the virus infection. For example, apoptosis early after IBDV infection restricts viral replication [40], while the occurrence of apoptosis in the later phase of IBDV infection is conducive to viral growth and spread [41]. It seems that apoptosis induced by gga-miR-16-5p during IBDV infection is skillfully manipulated by the virus to promote its replication. In addition, miR-16-5p-induced apoptosis can play an antiviral and anti-inflammatory role in other pathogenic infections. It was reported that enterovirus 71 (EV71) (the predominant pathogen of hand-foot-and-mouth disease (HFMD)) infection induces the expression of has-miR-16-5p, which promotes cell apoptosis through activating caspase-3 and inhibits EV71 replication [126]. Furthermore, gga-miR-16-5p was found to promote apoptosis of mycoplasma gallisepticum (MG)-infected DF-1 cells through directly targeting phosphoinositide-3-kinase regulatory subunit 1 (PIK3R1), subsequently inhibiting the PI3K/Akt/NF-κB pathway and exerting an anti-inflammatory effect. At the same time, the cell proliferation and cycle progression of MG-infected DF-1 cells were inhibited by gga-miR-16-5p [127].

Based on the experimental evidence available so far, miRNAs enhance viral replication in three ways: first, miRNA directly targets anti-apoptotic proteins to promote the release of virions (gga-miR-16-5p→Bcl2); second, miRNAs target IFN-related molecules to inhibit the production of an interferon and hijack the interferon-mediated antiviral pathway (gga-miR-142-5p→MDA5; gga-miR-9-3p→IRF2); and third, miRNA inhibits the p53-related antiviral signaling pathway (miR-2127-3p→p53) (Figure 1).

## 6. Conclusions

So far, 882 precursor miRNAs and 1232 mature miRNAs have been identified in chickens (http://www.mirbase.org/cgi-bin/browse.pl), but only a few have been characterized. Nine miRNAs have been found to affect IBDV infection by regulating apoptosis and/or I-IFN expression or binding to the viral genome. Among these miRNAs, gga-miR-130b-3p, gga-miR-454-3p, gga-miR-155-5p, gga-miR-21-5p and gga-miR-27b-3p inhibit IBDV infection, while gga-miR-2127-3p, gga-miR-142-5p, gga-miR-9-3p and gga-miR-16-5p promote IBDV replication. It was demonstrated that IBDV replication could be effectively inhibited by the anti-VP1 and anti-VP2 miRNAs delivered by recombinant avian adeno-associated virus (rAAAV) [128,129], suggesting the feasibility of miRNA as a novel antiviral drug. However, to date, the exact mechanism of initiating miRNA expressions following IBDV infection is still unraveled. It was found that the promoter regions of gga-mir-16-2 (one of the precursors of gga-miR-16-5p) and gga-mir-27b (the precursor of gga-miR-27b-3p) were demethylated after IBDV infection, which upregulates the expression of gga-miR-16-5p and gga-miR-27b-3p, respectively [22,26]. In addition to the epigenetic modification of the miRNA promoter, transcription factors, lncRNA/circRNA and histone modifications might also be important to regulate miRNA expressions. An elucidation of the regulatory mechanism for miRNA expressions in IBDV-infected cells will help to understand the pathogenesis of IBDV infection, and provide clues to the prevention and control of IBDV infection.
